# Low‐intensity focused ultrasound neuromodulation for drug‐resistant epilepsy: A randomized, sham‐controlled crossover trial

**DOI:** 10.1002/epi.70404

**Published:** 2026-08-01

**Authors:** Chien‐Chen Chou, Yen‐Cheng Shih, Yi‐Hsiu Chen, Po‐Tso Lin, Chun‐Fu Lin, Po‐Chun Chu, Hao‐Li Liu, Cheng‐Chia Lee, Hsiang‐Yu Yu

**Affiliations:** ^1^ Department of Neurology, Neurological Institute Taipei Veterans General Hospital Taipei Taiwan; ^2^ School of Medicine National Yang‐Ming Chiao Tung University Taipei Taiwan; ^3^ Brain Research Center National Yang‐Ming Chiao Tung University Taipei Taiwan; ^4^ Department of Neurosurgery, Neurological Institute Taipei Veterans General Hospital Taipei Taiwan; ^5^ Department of Electrical Engineering National Taiwan University Taipei Taiwan

**Keywords:** crossover trial, epilepsy, focused ultrasound, neuromodulation, noninvasive brain stimulation

## Abstract

**Objective:**

This study was undertaken to evaluate the safety, feasibility, and efficacy of low‐intensity focused ultrasound (LIFU) as a noninvasive neuromodulation technique in patients with drug‐resistant epilepsy (DRE).

**Methods:**

In this pilot, single‐blind, randomized sham‐controlled crossover trial, 12 patients with DRE underwent both LIFU and sham stimulation in a randomized sequence, targeting the seizure onset zone (SOZ), each followed by a 4‐week observation period. Seizure frequency was analyzed as proportional change from baseline using linear mixed‐effects models. An open‐label extension phase evaluated longitudinal seizure outcomes following LIFU. Safety assessments included neurological examinations, magnetic resonance imaging (MRI), and neuropsychological and quality of life (QOL) measures.

**Results:**

During the crossover phase, LIFU did not significantly reduce seizure frequency compared with sham (estimate = .49, 95% confidence interval [CI] = −.04 to 1.01, *p* = .068). No period or sequence effects were observed. Conversely, during the open‐label extension phase, seizure frequency demonstrated a significant longitudinal reduction following LIFU (β = −14.0 percentage points per month, 95% CI = −22.2 to −5.8, *p* = .001). Post‐LIFU MRI showed no structural lesions. No significant changes were observed in anxiety, depression, or QOL scores. There were only transient mild‐to‐moderate adverse events reported, without serious complications.

**Significance:**

LIFU neuromodulation targeting the SOZ is safe and well tolerated. Although the primary crossover analysis did not demonstrate a statistically significant antiseizure effect, delayed seizure reduction during the extended follow‐up suggests potential sustained neuromodulatory effects. Larger parallel‐group trials with longer observation periods are warranted.

**Key points**

- The first sham‐controlled crossover trial of LIFU in drug‐resistant epilepsy was conducted.
- LIFU targeting seizure onset zone was safe and well tolerated.
- The primary crossover analysis did not demonstrate a statistically significant antiseizure effect.
- Exploratory longitudinal follow‐up suggested delayed seizure reduction following LIFU treatment.

## 1 INTRODUCTION

Focused ultrasound (FUS) therapy for neurological disorders has advanced rapidly in recent years.^1^ With the development of phased‐array elements, the limitation of transcranial ultrasound energy transmission has been overcome, allowing ultrasound energy to be effectively delivered into the intracranial space. As a result, incisionless intracranial ablation and neuromodulation have become promising therapeutic options. Since the US Food and Drug Administration approved high‐intensity FUS for the treatment of essential tremor in 2016,^2^ and subsequently for tremor‐dominant Parkinson disease,^3^ the number of clinical trials in neurological disease using FUS has increased exponentially.

Approximately one third of patients with epilepsy are drug‐resistant. These patients experience significant negative impacts on quality of life, cognition, emotional well‐being, and even survival.^4^ Resective surgery can effectively achieve seizure freedom, but not all patients with drug‐resistant epilepsy (DRE) are suitable candidates for surgery.^5^ Neuromodulation is an alternative treatment option for DRE. Vagus nerve stimulation (VNS), deep brain stimulation (DBS), and responsive neurostimulation have been shown to reduce seizure frequency; however, these approaches require surgical implantation of devices. Although implantation‐related risks such as infection and hemorrhage are relatively low, they remain important considerations in clinical decision‐making.^6^

Noninvasive neuromodulation techniques, including transcranial magnetic stimulation^7^ and transcranial direct current stimulation,^8^ have also been applied for epilepsy treatment. Although some studies have reported potential benefits, the level of evidence remains limited. In addition, these techniques lack sufficient spatial precision and cannot effectively target deep brain structures involved in epileptic networks.

Transcranial low‐intensity focused ultrasound (LIFU) possesses several advantages over existing neuromodulation techniques, including high spatial precision, the ability to target deep brain structures, and noninvasive delivery. Preclinical studies have demonstrated that focused ultrasound can modulate epileptic activity and neural excitability.^9^, ^10^, ^11^ Our previously published phase 1 study further confirmed the feasibility and safety of LIFU neuromodulation in patients with epilepsy.^12^ However, clinical evidence from randomized controlled trials remains limited. Therefore, we conducted a single‐blind, sham‐controlled, crossover trial to investigate the safety and efficacy of transcranial focused ultrasound neuromodulation in patients with DRE. We hypothesized that LIFU would reduce seizure frequency compared with sham stimulation.

## 2 MATERIALS AND METHODS

### 2.1 Patients

This pilot, single‐blind, randomized, sham‐controlled crossover study was conducted at Taipei Veterans General Hospital between January 2022 and March 2024.

Eligible participants were adults aged ≥20 years with DRE, defined as failure of at least three antiseizure medications. Although this criterion was more stringent than the International League Against Epilepsy definition of DRE, a higher threshold was selected because LIFU represented a novel investigational intervention, and enrollment was therefore restricted to patients with particularly severe and highly treatment‐refractory epilepsy. Participants were also required to have a clearly localized seizure onset zone (SOZ) determined by comprehensive presurgical evaluation, including clinical semiology, magnetic resonance imaging (MRI), fluorodeoxyglucose positron emission tomography, video‐electroencephalographic (EEG) monitoring, neuropsychological assessment, and intracranial EEG when indicated.

Participants were required to have stable antiseizure medication regimens and at least three countable seizures during the 28‐day baseline period.

Key exclusion criteria included unstable medical or psychiatric conditions, contraindications to focused ultrasound or MRI, recent epilepsy‐related interventions (radiofrequency thermocoagulation, VNS, dosing change, or resective surgery), pregnancy, or any condition that could interfere with study participation. Detailed eligibility criteria are provided in Table S1.

The study was approved by the institutional review board (2021–05‐006A) and registered at ClinicalTrials.gov (NCT04999046). All participants provided written informed consent. This study was funded by the NaviFUS Corporation, with involvement limited to technical support. Although this was an industry‐sponsored study, the principal investigator and coinvestigators maintained full authority over all aspects of the study, including the study design, patient enrollment, patient outcome assessment, and data management.

### 2.2 Randomization and masking

Participants were randomized in a 1:1 ratio to receive either FUS followed by sham treatment or sham followed by FUS using a computer‐generated randomization sequence. Participants were blinded to treatment allocation, whereas investigators performing the intervention were not blinded due to the nature of the procedure.

### 2.3 Study design

Each participant underwent two treatment phases, consisting of one FUS session and one sham session, separated by a 4‐week (30 ± 7 days) observation period.

Seizure frequency was recorded using daily seizure diaries throughout the study.

Baseline seizure frequency for crossover analysis was defined as the mean seizure frequency during the 28 days preceding the first treatment.

After completion of the crossover phase, participants were invited to enter an open‐label extension phase for longitudinal follow‐up (Figure 1).

**FIGURE 1:**
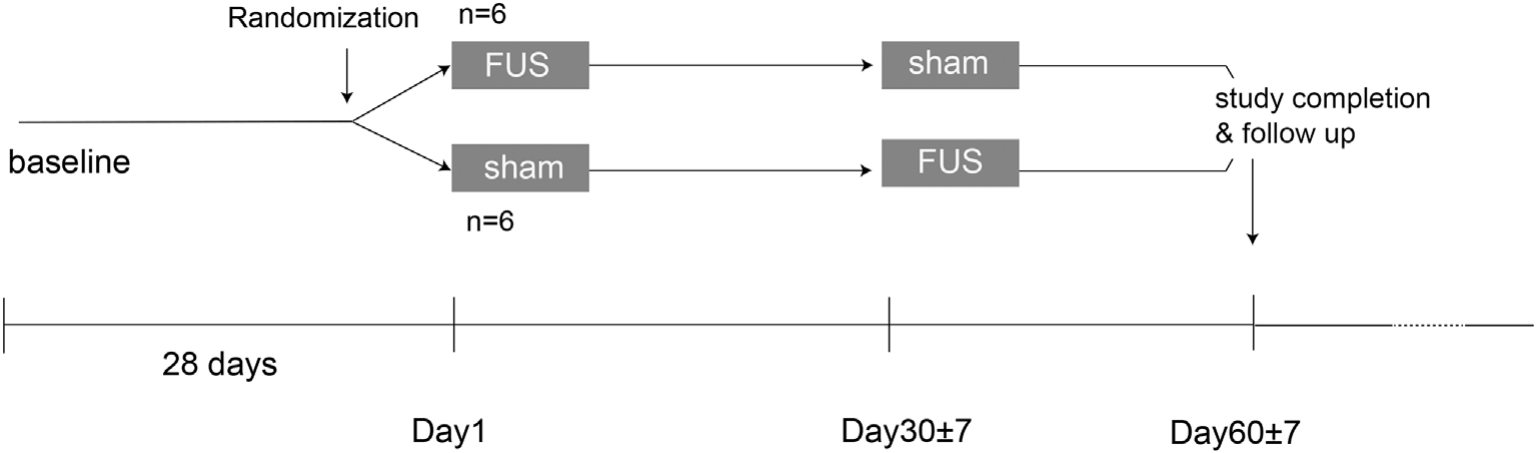
Study design. After a 28‐day baseline period, participants were randomized to low‐intensity focused ultrasound (LIFU)–sham or sham–LIFU sequences, with crossover after 4‐week treatment phases. An exploratory open‐label extension phase followed. FUS, focused ultrasound.

### 2.4 LIFU treatment target, planning, and delivery

The treatment device used in this study was provided by NaviFUS Corporation, a 256‐element phase array LIFU, guided by a navigation system. The SOZ was selected as the treatment target.

Sonication was simulated using the NaviFUS onboard software. To ensure acoustic delivery, the attenuation of acoustic pressure during cranial bone penetration was estimated from pretreatment computed tomography (CT) images and calculated within the NaviFUS system. Skull properties were extracted from these CT scans, where the software automatically segmented the skull structure based on Hounsfield unit (HU) thresholds. The skull thickness was defined as the Euclidean distance between the outer and inner tables along the planned trajectory, and the skull density ratio was parameterized as the ratio of mean HU values between the marrow and cortical bone. These parameters allowed the simulation algorithm to estimate in situ intensity by accounting for both transcranial transmission loss and soft‐tissue attenuation.

In addition to preoperative planning, the spatial distribution and peak intensity of the delivered transcranial acoustic energy were quantified through post hoc simulation analysis utilizing a cross‐validation approach that combined the NaviFUS software with the open‐source k‐Wave acoustics toolbox. This analysis accounted for skull‐induced beam attenuation and deformation, providing a quantified readout of the pressure deposited at the anatomical target. This approach enabled the verification of focal displacement and peak transcranial mechanical index (MI_tc) based on the physical properties of the skull and the specific sonication parameters.

After determining the treatment targets, several potential trajectories were built. Among them, the optimal trajectory was selected according to penetration energy, the number of orthogonal elements, and the estimated temperature rise. The operating procedure of the device was similar to that of our previous human trial, using a NaviFUS system compatible with the StealthStation S8 neuronavigation system (Medtronic).^12^

At the scalp site where the transducer contacted the treatment trajectory, a circular area approximately 10 cm in diameter was shaved. The patient remained awake and was seated in an adjustable reclining chair, with the position adjusted to ensure comfort throughout the procedure. The treatment could be terminated immediately if the patient experienced intolerable discomfort at any time during the procedure.

Acoustic parameters were defined at both safety ceiling and therapeutic levels. The safety ceiling was set at an estimated intracranial spatial‐peak temporal‐average intensity (I_SPTA_) of 2.8 W/cm^2^, consistent with the maximum output validated in our prior first‐in‐human study.^12^ At a center frequency of 500 kHz, this corresponded to an intracranial mechanical index (MI) of .75, peak pressure of .53 MPa, and root‐mean‐square (RMS) pressure of .37 MPa.

The planned therapeutic intensity was set at an intracranial I_SPTA_ of .3 W/cm^2^ (MI = .25), corresponding to a peak pressure of .18 MPa and RMS pressure of .12 MPa. Treatment trajectories were selected to achieve estimated energy deposition as close as possible to the predefined therapeutic level.

Ultrasound was delivered using a burst length of 3 ms, duty cycle of 30%, and pulse repetition frequency of 100 Hz. Each session consisted of three consecutive 5‐min sonications separated by two 5‐min intermissions. These stimulation parameters were selected based on our previous preclinical studies demonstrating suppression of epileptiform activity without tissue injury and were subsequently adopted in our pilot first‐in‐human study of LIFU neuromodulation in DRE.^10^, ^11^, ^12^

Sham treatment followed an identical treatment workflow, including scalp shaving, patient positioning, neuronavigation‐guided target localization, transducer placement, treatment duration, and operator–patient interactions. No ultrasound energy was delivered (I_SPTA_ = 0 W/cm^2^). To preserve blinding, prerecorded background sounds simulating active sonication were played throughout the procedure. Participants remained unaware of treatment allocation and were informed that sensory experiences during treatment could vary between individuals. Both active and sham procedures required approximately 25 min and were conducted in an identical treatment environment.

### 2.5 Assessment of safety and efficacy

Safety and efficacy were evaluated using standardized clinical and patient‐reported outcome measures. Participants completed the Quality of Life in Epilepsy Inventory‐31 (QOLIE‐31),^13^ Beck Anxiety Inventory (BAI),^14^ and Beck Depression Inventory (BDI)^15^ at baseline, after FUS and sham treatments, and at the end of the study. Comprehensive neurological examinations were performed before and after each intervention. Brain MRI was obtained 14 days after LIFU treatment.

After completion of the initial observation phase of the crossover design, we extended the follow‐up period to further monitor seizure outcomes in the participants. During this extended observation period, follow‐up and data collection were discontinued if there were any changes in the antiseizure medication regimen, if the participant underwent epilepsy‐related surgical intervention, or if adjustments were made to the stimulation parameters of VNS.

All treatment‐related discomforts and adverse events occurring during the intervention and follow‐up periods were systematically recorded. The principal investigator determined their potential relationship to the study intervention. Seizure frequency analyses included all habitual seizures, regardless of seizure type or severity.

### 2.6 Statistical analysis

#### 2.6.1 Crossover phase analysis

Given the substantial variability in baseline seizure frequency across participants, seizure outcomes were normalized to improve statistical stability. For the short time crossover analysis, the baseline seizure count was defined as the average seizure number in the past 28 days. The proportional change in seizure frequency was calculated by subtracting the mean posttreatment seizure count from the baseline value and dividing by the baseline count. This approach minimized data dispersion and enhanced the robustness of subsequent analyses.

For the short‐term, single‐blind, sham‐controlled crossover phase (4 weeks per arm), data were analyzed using linear mixed‐effects models. Treatment, period, and sequence were included as fixed effects, and a random intercept was specified for each participant. A variance component covariance structure was adopted to account for within‐subject correlations. Model parameters were estimated using restricted maximum likelihood. Sensitivity analyses were performed using the nonparametric approach for 2 × 2 crossover designs described by Hills and Armitage,^16^ in which treatment differences were evaluated according to period and sequence (FUS–sham vs. sham–FUS). In addition, direct within‐subject comparisons of seizure frequency between the two treatment arms were conducted using the Wilcoxon signed‐rank test.

#### 2.6.2 Secondary outcomes of the crossover study phase

The scores of BAI, BDI, and QOLIE‐31 obtained at the end of each phase were obtained. Changes from baseline were analyzed using linear models including treatment, period, and sequence as fixed effects. Sensitivity analyses using nonparametric methods yielded consistent results.

#### 2.6.3 Extended time followed up

Following completion of the crossover phase, an open‐label extension was conducted to evaluate the longer term effects of LIFU.

Longitudinal analyses were performed to assess changes in seizure frequency over time after LIFU treatment. As follow‐up duration varied across participants and repeated measurements were collected at multiple time points, linear mixed‐effects modeling was used to accommodate unbalanced data and missing observations without requiring complete‐case analysis.

The models included time since FUS initiation as a fixed effect and subject‐specific random intercepts to account for within‐subject correlations. A Gaussian error distribution was assumed, and parameters were estimated using restricted maximum likelihood.

Seizure outcomes were expressed as proportional changes from baseline, defined as the mean 28‐day seizure frequency during the 3 months preceding treatment initiation. For participants in the sham‐first sequence, the sham treatment month was included as part of the baseline period to align the starting point of LIFU exposure across individuals.

To evaluate the robustness of the findings, a sensitivity analysis was performed using a restricted dataset limited to the first 5 months of follow‐up, during which all participants had complete outcome data. The same mixed‐effects modeling framework was applied.

All seizure frequency data were analyzed as percentage changes relative to baseline. Statistical significance was defined as a two‐tailed *p*‐value < .05. All analyses were conducted using SPSS version 26.

## 3 RESULTS

### 3.1 Patients

Twelve patients were randomized and completed both treatment phases. The clinical characteristics are summarized in Table 1.

**TABLE 1:** Patients' demographics and epilepsy‐related information.

Case	Gender	Age, years	Age at onset of epilepsy, years	Seizure patterns	Brain MRI findings	Target of treatment
1	F	44	25	FIC	Normal	L't hippocampus
2	M	33	17	FIC	R't T s/p tumor resection	R't hippocampus
3	F	25	10	FPC	R't FC encephalomalacia	R't FC
4	M	56	44	FIC	L't MTS	L't hippocampus
5	M	32	19	FIC	Normal	L't hippocampus
6	M	60	29	FIC	R't MTS	R't hippocampus
7	F	50	23	FIC	L't medial T cavernoma	L't hippocampus
8	F	33	15	FPC/FIC	Normal	L't temporal operculum
9	F	47	12	FPC	Left P FCD s/p RFTC	L't P
10	F	37	7	FIC	L't medial Fr s/p resection	L't medial frontal
11	M	21	1.5	FIC	L't MTS	L't hippocampus
12	M	29	.9	FIC	L't MTS	L't hippocampus

Abbreviations: F, female; FC, frontocentral; FCD, focal cortical dysplasia; FIC, focal impaired consciousness seizures; FPC, focal preserved consciousness seizures; Fr, frontal; L't, left; M, male; MRI, magnetic resonance imaging; MTS, mesial temporal sclerosis; P, parietal; RFTC, radiofrequency thermocoagulation; R't, right; s/p, status post; T, temporal lobe.

### 3.2 Individualized treatment parameters and acoustic simulation

The patient‐specific treatment parameters and acoustic simulation data are summarized in Table 2. The distance from the scalp to the target region varied considerably among the 12 participants, ranging from 31 mm (Case 9) to 98 mm (Case 6), with an average of 62.5 ± 25.9 mm. Based on the preoperative imaging, the simulated transcranial acoustic penetration rate ranged from 19.53% to 36.6%, with an average of 27.2% ± 5.9%.

**TABLE 2:** Data of output and estimated energy of the study subjects.

Case	Scalp to target, mm	Output I_SPTA_, W/cm^2^	Output MI	Estimated transcranial I_SPTA_, W/cm^2^	Estimated transcranial MI_tc	Penetration rate, %	Focal displacement values			
							dx, mm	dy, mm	dz, mm	d, mm
1	50	7.2	1.2	.3	.24	20.4	4	1	3	5.10
2	96	5	1	.33	.26	25.9	5	1	24	24.50
3	33	3.2	.8	.36	.27	33.6	3	0	2	3.61
4	87	5	1	.34	.26	26.2	1	0	1	1.41
5	55	7.2	1.2	.27	.23	19.5	1	4	7	8.12
6	98	1.8	.6	.24	.22	36.6	1	1	1	1.73
7	97	3.2	.8	.34	.26	32.8	4	0	13	13.60
8	32	5	1	.26	.23	22.8	0	1	2	2.24
9	31	1.8	.6	.24	.22	36.5	3	3	2	4.69
10	37	7.2	1.2	.33	.26	21.5	1	1	3	3.32
11	77	5	1	.35	.27	26.6	0	0	4	4.00
12	57	5	1	.29	.24	24.0	0	1	8	8.06
Avg.	62.5 ± 25.9	4.7 ± 1.8	1.0 ± .2	.30 ± .04	.25 ± .02	27.2 ± 5.9	1.9 ± 1.7	1.1 ± 1.2	5.8 ± 6.4	6.7 ± 6.3

*Note*: dx, dy, dz: translational deviations (mm) along the x‐, y‐, and z‐axes, respectively.

Abbreviations: Avg., average; d, three‐dimensional Euclidean distance between the planned target and the estimated acoustic focus; I_SPTA_, intracranial spatial‐peak temporal‐average intensity; MI, mechanical index; MI_tc, transcranial mechanical index.

To achieve sufficient energy delivery for neuromodulation, the system output I_SPTA_ was set between 1.8 W/cm^2^ and 7.2 W/cm^2^ (4.7 ± 1.8 W/cm^2^), corresponding to an output MI ranging from .6 to 1.2 (1.0 ± .2), respectively. Cases 1, 5, and 10 required a higher system output (I_SPTA_ = 7.2 W/cm^2^, output MI = 1.2) to compensate their relatively low penetration rates (20.4%, 19.53%, and 21.45%, respectively). After accounting for acoustic attenuation by the skull, the estimated in situ energy reaching the focal targets yielded an estimated transcranial I_SPTA_ ranging from .24 to .36 W/cm^2^ (.3 ± .04 W/cm^2^), and an estimated MI_tc ranging from .22 to .27 (.25 ± .02). Post hoc acoustic simulations were conducted for all 12 cases, and the corresponding results are summarized in Table 2. The estimated targeting error, defined as the three‐dimensional Euclidean distance between the planned target and the simulated acoustic focus, ranged from 1.41 mm to 24.50 mm, with a mean of 6.7 ± 6.3 mm. A representative acoustic beam pattern illustrating the spatial distribution of the simulated focus is shown in Figure 2.

**FIGURE 2:**
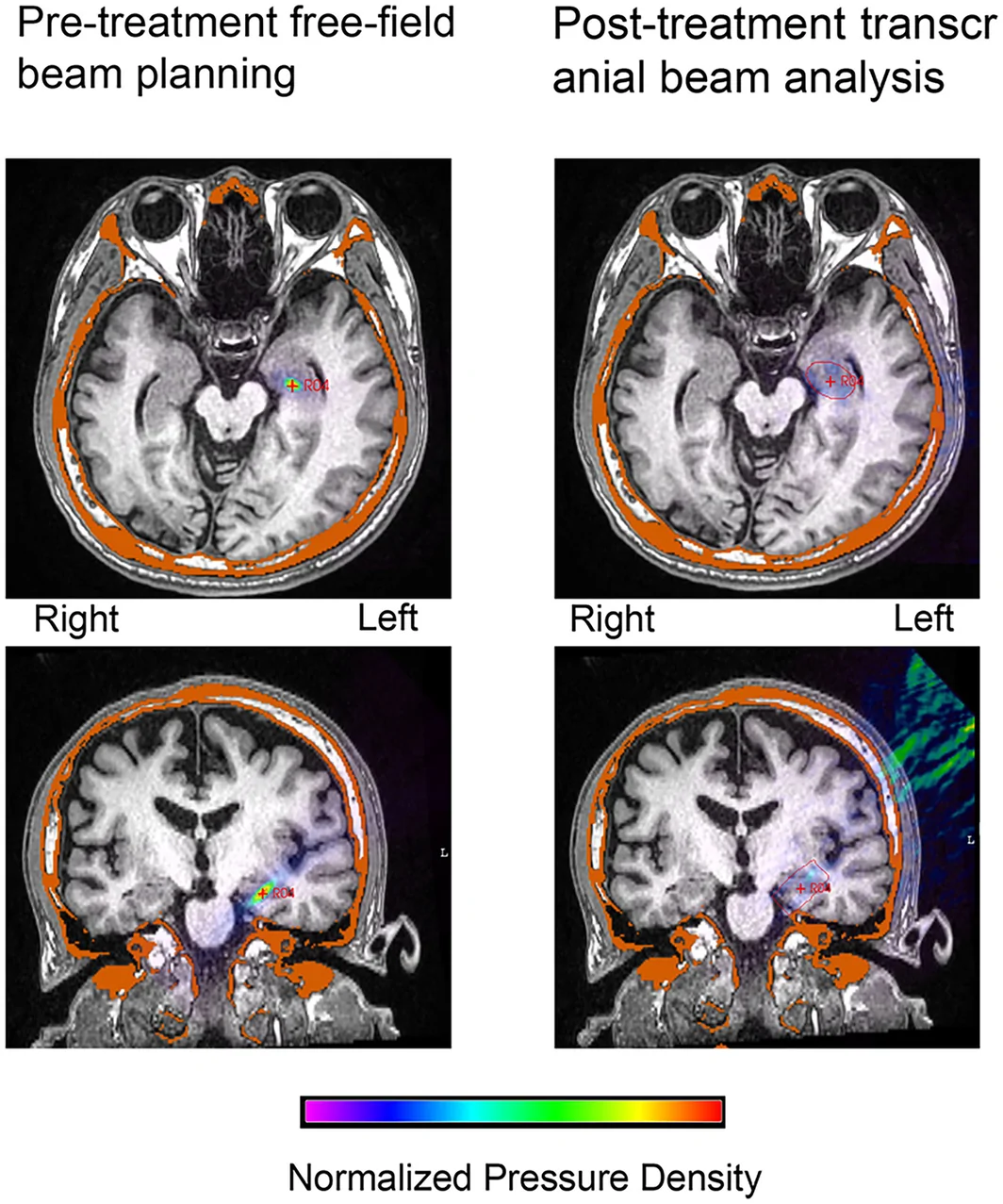
Pretreatment free‐field beam planning and posttreatment transcranial beam analysis. Axial (top) and coronal (bottom) magnetic resonance imaging views illustrate the targeting of the seizure onset zone. Left panels show the presonication planning, with the red crosshair denoting the intended focus. Right panels display the posttreatment transcranial simulation analysis using NaviFUS software to account for transcranial focal beam attenuation and deformation. The normalized pressure density map shows a slight inward focal displacement due to skull refraction; however, the peak intensity, quantified by the transcranial mechanical index, remains strictly confined within the predefined target volume. In this case, approximately 76% of the acoustic pressure was attenuated during transcranial sonication.

### 3.3 Primary outcome: Crossover phase

During the initial 8‐week crossover phase, 12 patients were randomized to receive either FUS followed by sham (*n* = 6) or sham followed by FUS (*n* = 6).

The primary crossover analysis using linear mixed‐effects modeling did not demonstrate a statistically significant difference between FUS and sham treatments (estimate = .49, 95% confidence interval [CI] = −.04 to 1.01, *p* = .068). This prespecified analysis reflects the entire crossover phase rather than the initial parallel‐group comparison during the first treatment period. No significant period or sequence effects were observed (both *p* > .4; Figure 3).

**FIGURE 3:**
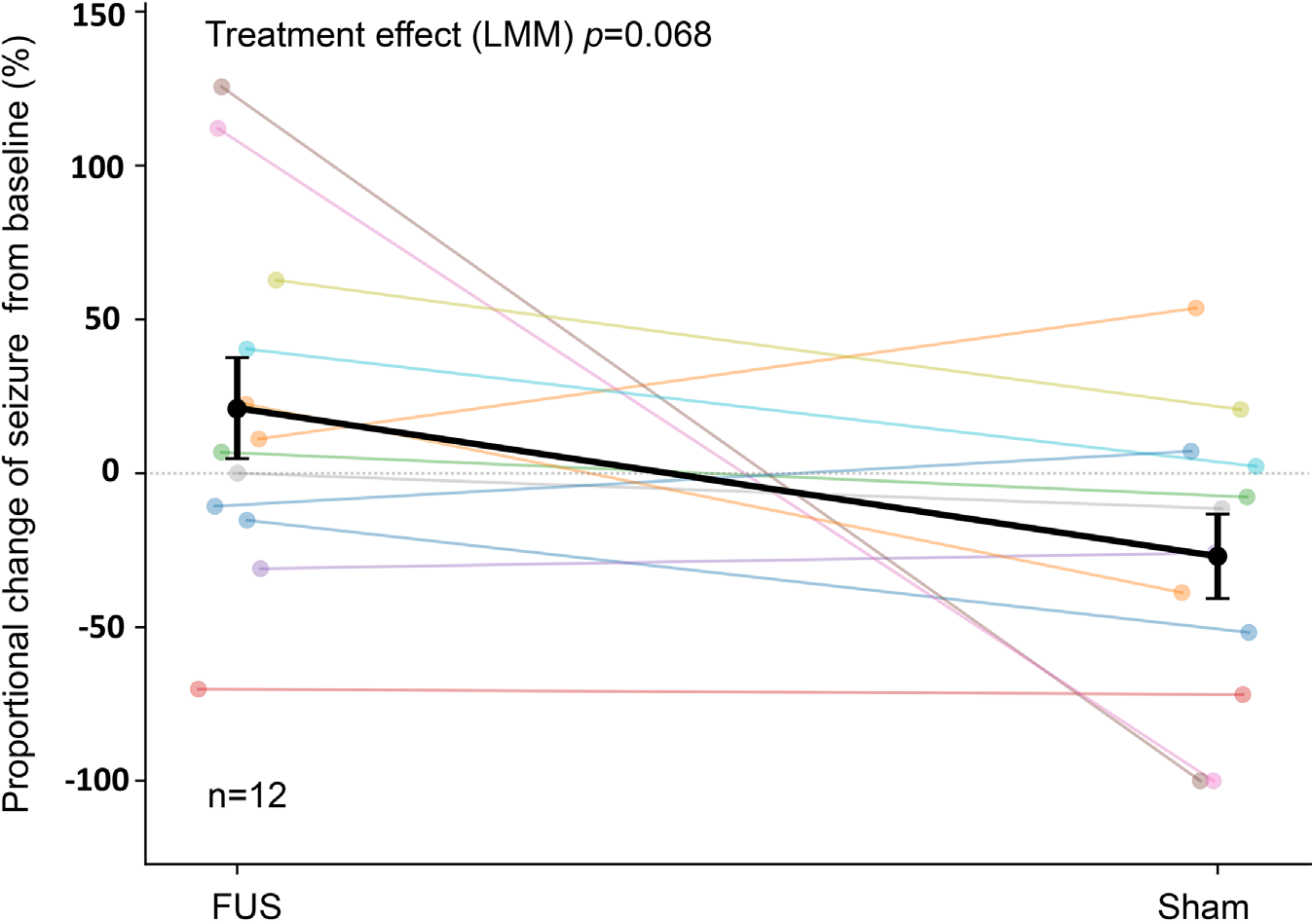
Seizure frequency change during the sham‐controlled crossover phase. Proportional change in seizure frequency relative to baseline during focused ultrasound (FUS) and sham treatment is shown. Each line represents an individual participant, and black circles represent mean ± SEM. Positive values indicate seizure worsening. No significant treatment effect was observed (linear mixed‐effects model [LMM], *p* = .068).

Sensitivity analyses using the nonparametric Hills–Armitage method yielded consistent results, with no significant carryover effect (*p* = .31). Direct within‐subject comparisons using the Wilcoxon signed‐rank test showed similar directional trends (*p* = .084). Sequence‐specific seizure trajectories throughout the original study timeline are presented in Figure 4.

**FIGURE 4:**
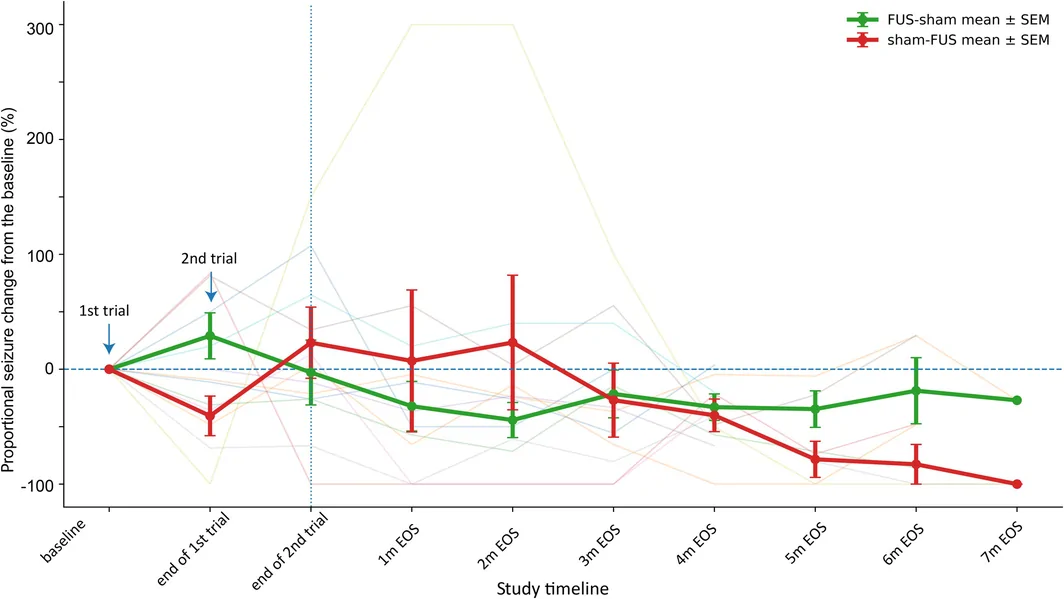
Sequence‐specific seizure trajectories across the original study timeline. Individual participant trajectories are shown as thin lines, whereas thick lines with error bars represent the group mean ± SEM. Participants were randomized to either the focused ultrasound (FUS)–sham or sham–FUS sequence. The x‐axis follows the original study timeline, including baseline, first treatment period (1st trial), second treatment period (2nd trial), and monthly follow‐up assessments after completion of the crossover phase (end‐of‐study [EOS] follow‐up). The vertical dotted line indicates the transition from the crossover phase to the EOS follow‐up. Negative values indicate seizure reduction relative to baseline. This figure illustrates the temporal evolution of seizure outcomes according to treatment sequence during the randomized crossover trial and facilitates interpretation of the immediate and delayed changes in seizure frequency. m, months.

### 3.4 Safety

No serious adverse events were observed. Treatment‐emergent adverse events were categorized according to their occurrence during sonication and during the posttreatment follow‐up period and are summarized in Tables 3 and 4. Mild headache was the most commonly reported adverse event and occurred during both FUS and sham treatment, as well as during follow‐up. Other transient symptoms included tremor (*n* = 1), dental vibration sensation (*n* = 1), and abdominal discomfort (*n* = 1). All adverse events were self‐limited and resolved spontaneously without medical intervention.

**TABLE 3:** Treatment‐emergent adverse events during sonication.

Case	Treatment	Adverse event	Severity	Outcome
1	FUS	Localized headache at the treatment site	Moderate	Resolved immediately after treatment
1	Sham	Soreness, eyebrow, the side of treatment	Mild	Resolved immediately after treatment
3	Sham	Scalp pain while being touched	mild	Resolved in hours
8	FUS	Upper molar vibration sensation	Mild	Resolved immediately after treatment
10	FUS	Localized headache at the treatment site	Mild	Resolved immediately after treatment
11	FUS	Neck soreness	Mild	Resolved immediately after treatment
11	Sham	Neck soreness	Mild	Resolved immediately after treatment

Abbreviation: FUS, focused ultrasound.

**TABLE 4:** Treatment‐emergent adverse events after sonication and during follow‐up.

Case	Treatment	Adverse effect	Onset	Severity	Outcome
1	FUS	Headache	Day 1, day 3, day 4, day 19, day 27	Mild	Each episode resolved spontaneously within 1 h; no recurrence after 1 month
1	FUS	Tremor, generalized	Day 6	Mild	Resolved spontaneously within minutes
1	Sham	Headache	Day 5, day 6	Mild	No recurrence after 1 week
7	Sham	Ice pick headache	Day 21	Mild	No recurrence after 1 week
8	Sham	Intermittent headache	Day 1–28, episode lasting ~5 min	Mild	Returned to baseline frequency within 4 weeks
8	Sham	Mild increase in lower abdominal discomfort	Day 6	Mild	Resolved within several days
6	FUS	Transient increase in seizure frequency (brief nonmotor seizures with impaired awareness)	Month 1–2	Mild	No injuries or falls; seizure frequency subsequently improved, and patient became seizure‐free after month 3

Abbreviation: FUS, focused ultrasound.

### 3.5 Brain MRI after the treatment

Structural brain MRI 2 weeks after the LIFU treatment showed no lesioning effect at the treatment targets of all the subjects.

### 3.6 Psychological and quality of life outcomes

No significant differences were observed between FUS and sham conditions in changes of anxiety, depression, or quality of life scores including total score and cognition‐related domain scores (Table 5).

**TABLE 5:** Results of LMM model analysis of the BAI, BDI, and total score and cognitive domain of QOLIE‐31.

Outcome (Δ from baseline)	Estimate (FUS–sham)	Standard error	95% CI	*p*
BAI change	3.83	2.72	−1.84 to 9.50	.174
BDI change	2.08	1.58	−1.21 to 5.38	.202
QOLIE‐31 change	.00	6.78	−14.15 to 14.15	1.000
Cognitive domain of QOLIE‐31	−4.30	5.59	−15.95 to 7.35	.450

*Note*: Estimates represent adjusted mean differences between focused ultrasound and sham conditions.

Abbreviations: BAI, Beck Anxiety Inventory; BDI, Beck Depression Inventory; CI, confidence interval; LMM, linear mixed‐effects model; QOLIE‐31, Quality of Life in Epilepsy Inventory‐31.

### 3.7 Exploratory open‐label extension phase

Follow‐up duration during the extended period varied across participants (9 months, *n* = 3; 8 months, *n* = 3; 7 months, *n* = 2; 6 months, *n* = 2; 5 months, *n* = 2), with all 12 participants completing at least 5 months of follow‐up. During the exploratory open‐label extension phase, linear mixed‐effects modeling demonstrated a significant longitudinal decrease in proportional seizure frequency (*β* = −14.0 percentage points/month, 95% CI = −22.2 to −5.8, *p* = .001). Despite early fluctuations, seizure reduction was overall sustained during follow‐up (Figures 4 and 5).

**FIGURE 5:**
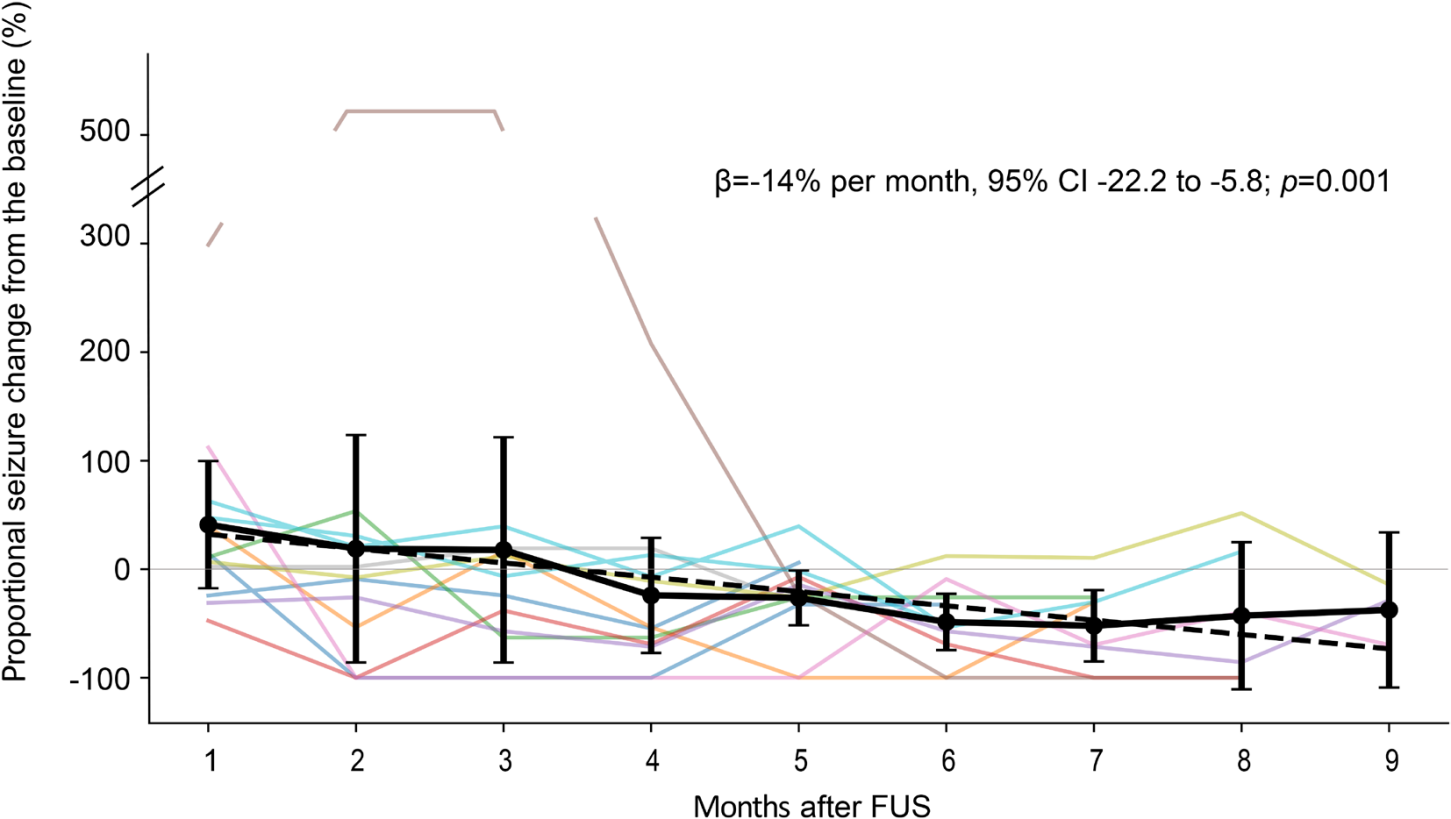
Longitudinal seizure outcomes aligned to the timing of focused ultrasound (FUS) treatment. Proportional seizure frequency changes are aligned to the timing of active FUS treatment for each participant, irrespective of treatment sequence. Thin lines represent individual participants, and the solid black line with error bars represents the group mean ± 95% confidence interval (CI). The dashed line indicates the fitted linear mixed‐effects model. The exploratory longitudinal analysis demonstrated a significant decrease in seizure frequency over time (*β* = −14.0 percentage points/month, 95% CI = −22.2 to −5.8, *p* = .001).

In the sensitivity analysis restricted to the first 5 months of follow‐up, all 12 participants contributed complete longitudinal data. The overall pattern remained consistent with the primary extended follow‐up analysis (*β* = −17.79 percentage points/month, 95% CI = −31.29 to −4.28, *p* = .011), supporting the extended‐period trend not being solely attributable to missing observations in later follow‐up months or to unequal follow‐up duration across participants.

### 3.8 Subgroup analysis

Patients were stratified into medial temporal lobe epilepsy (*n* = 8) and non‐medial temporal lobe epilepsy (*n* = 4) groups. Subgroup analysis did not demonstrate a significant difference in longitudinal trends between mesial and extramesial temporal epilepsy (*p* = .461).

## 4 DISCUSSION

In this sham‐controlled crossover study, the primary outcome analysis did not demonstrate a statistically significant difference in seizure reduction between LIFU and sham stimulation during the initial 1‐month observation period. However, exploratory extended follow‐up revealed a gradual reduction in seizure frequency relative to baseline, with more consistent improvement emerging after the second month. Importantly, LIFU was well tolerated, with no serious adverse events observed. These findings support the safety and feasibility of LIFU neuromodulation in patients with DRE and provide preliminary evidence supporting the possibility of delayed neuromodulatory effects.

Clinical evidence for LIFU neuromodulation in epilepsy remains limited. Stern et al. reported MRI‐guided LIFU in mesial temporal lobe epilepsy with favorable safety outcomes.^17^ Similarly, Bubrick et al. demonstrated seizure reduction following LIFUS, with a mean reduction of approximately 50% over 6 months.^18^ Consistent with our previously reported six‐patient pilot study, LIFU treatment was generally well tolerated, with only mild transient headache reported and no serious treatment‐related adverse events. One subject developed transient functional neurological symptoms in the context of marked seizure reduction, which was considered consistent with forced normalization.^12^ Our present findings further extend the existing literature by providing sham‐controlled safety data. Furthermore, whereas prior studies primarily targeted the hippocampus, extratemporal targets were included in our cohort, suggesting that the neuromodulatory application of LIFU may extend beyond mesial temporal structures.

Although no significant short‐term effect was observed, seizure reduction became apparent during extended follow‐up. This delayed response is consistent with the time course observed in established neuromodulation therapies such as VNS and DBS,^6^ where therapeutic effects typically emerge over months. However, unlike these continuous stimulation paradigms, the observation that a single FUS session may induce prolonged seizure reduction raises important mechanistic considerations.

Preclinical studies provide potential explanations for this phenomenon. FUS has been shown to induce both immediate and sustained modulation of neuronal activity.^19^ In epilepsy models, FUS suppresses acute seizure activity^9^, ^10^ and reduces epileptiform discharges for several weeks following a single treatment.^11^ These findings suggest that FUS may induce longer lasting changes in network excitability, potentially through synaptic plasticity, ion channel modulation, or network‐level reorganization.

Despite these encouraging findings, the optimal stimulation parameters for epilepsy neuromodulation remain to be established. Heterogeneity in clinical outcomes likely arises from variability in stimulation protocols, target selection, and treatment schedules across studies. In the present study, post hoc transcranial simulations demonstrated displacement of the acoustic focal point relative to the planned target coordinates (Figure 2 and Table 2). A relatively large focal displacement was observed in two specific participants, Case 2 (24.5 mm) and Case 7 (13.6 mm). Both subjects presented with relatively deep target locations (96 mm and 97 mm, respectively), suggesting that the combination of increased target depth and individual skull geometry may have compounded the magnitude of the focal deviation. Although the simulated acoustic pressure distribution suggested adequate target engagement, displacement of the focal maximum from the core epileptogenic zone may have reduced the biological effect of stimulation and contributed to interindividual variability in clinical response.

Accurate and consistent energy delivery to the intended target is therefore critical and may increase the treatment effect. However, the post hoc analytical approach applied in this study does not provide real‐time intraoperative feedback. The integration of real‐time monitoring techniques, such as acoustic emission mapping (AEM),^20^ into future clinical protocols would be of considerable value. AEM enables dynamic adjustment of focal positioning during treatment to compensate for acoustic distortion caused by skull refraction and tissue heterogeneity, thereby improving targeting precision.

Furthermore, considering the substantial anatomical extent and complex geometry of structures such as the hippocampus, a single‐target approach may provide limited spatial coverage. Future strategies incorporating multitarget trajectories or volumetric sonication, guided by rapid AEM feedback, may enhance coverage of broader epileptogenic regions and ultimately improve both neuromodulatory efficacy and long‐term clinical outcomes.

Although treatment planning, neuronavigation guidance, and post hoc acoustic simulations provided information regarding the spatial accuracy and estimated acoustic exposure at the target, the present study did not include a direct biomarker of neuromodulatory target engagement. Whereas previous human intracranial EEG studies from our group demonstrated localized electrophysiological changes following LIFU sonication, including reductions in low‐frequency power within the sonicated region, no such physiological measurements were incorporated into the current trial.^12^ Consequently, the relationship between acoustic delivery, target engagement, and clinical response could not be directly assessed. At present, no validated target engagement measure exists for LIFU neuromodulation in epilepsy, although electrophysiological, imaging, and network‐based biomarkers are actively being investigated. Future studies incorporating objective measures of target engagement will be important for optimizing treatment parameters and clarifying the relationship between acoustic delivery, biological response, and clinical outcomes.

Safety remains a critical consideration in FUS neuromodulation. Treatment parameters and target selection are closely associated with safety profiles. A recent report described brain injury in a patient undergoing FUS neuromodulation for substance use disorder, underscoring the importance of parameter optimization.^21^ In response, experts in the field have proposed a framework distinguishing high‐, intermediate‐, and low‐intensity FUS applications, and have suggested that low‐intensity protocols with an MI_tc < 1.9 fall within a safe range.^22^ The parameters employed in the present study were within this recommended safety threshold. In addition, a high proportion of participants underwent hippocampal sonication in the present study. Given previous reports of cognitive changes following high‐intensity focused ultrasound lesioning of the anterior nucleus of the thalamus, continued cognitive monitoring remains warranted even in low‐intensity neuromodulation protocols.^23^ Although formal memory testing was not incorporated into the study design, no evidence of cognitive deterioration was observed on the patient‐reported cognitive domain of the QOLIE‐31, and no significant differences were detected between the FUS and sham conditions. These findings provide preliminary reassurance regarding the cognitive safety of LIFU, although future studies should include comprehensive neuropsychological assessments, particularly of memory function, to better characterize potential cognitive effects.

### 4.1 Limitations

Several limitations should be acknowledged. First, this was a small exploratory crossover study conducted during the early phase of clinical translation. The sample size was limited, and the study was not powered to detect modest treatment effects or permit meaningful subgroup analyses. When the trial was designed, clinical experience with LIFU neuromodulation for epilepsy was extremely limited, and the study was therefore intentionally conceived as a feasibility study to evaluate safety, feasibility, and preliminary efficacy signals. Consequently, the findings should be interpreted cautiously and require confirmation in larger, adequately powered clinical trials.

Second, although the sham procedure incorporated background sounds simulating active sonication to facilitate blinding, the sensory experience associated with ultrasound delivery could not be fully replicated when no acoustic energy was delivered. Therefore, some participants may have been able to speculate about treatment allocation. However, transient discomfort and adverse effects were also reported during and after sham treatment, suggesting that subjective sensations alone were not reliable indicators of treatment assignment.

Third, the crossover design may have been affected by an insufficient washout duration, particularly given the delayed neuromodulatory effects observed in the extended follow‐up analysis. This may have reduced the ability to detect differences between active and sham conditions. Accordingly, the treatment comparisons during the crossover phase, including the apparent numerical advantage of sham during the first treatment period, should be interpreted with caution, because delayed carryover effects cannot be excluded.

Fourth, heterogeneity in stimulation targets may have contributed to variability in treatment response and limited the ability to identify patient subgroups most likely to benefit from LIFU neuromodulation.

Finally, the exploratory extension phase was not sham‐controlled, because all participants ultimately received active treatment during the crossover study. Therefore, the observed long‐term seizure reduction should be considered hypothesis‐generating and cannot be definitively attributed to LIFU treatment.

## 5 CONCLUSIONS

This pilot sham‐controlled crossover study demonstrates that LIFU neuromodulation is safe and well tolerated in patients with DRE. Although the primary crossover analysis did not demonstrate a statistically significant treatment effect, exploratory longitudinal follow‐up suggested a potential delayed neuromodulatory effect associated with progressive seizure reduction over time. These findings support the feasibility of LIFU as a noninvasive neuromodulation approach for epilepsy and provide important preliminary data for future clinical development. Larger randomized parallel‐group trials with longer follow‐up periods will be necessary to confirm efficacy, identify optimal treatment parameters, and better characterize the temporal profile of treatment response.

## AUTHOR CONTRIBUTIONS

**Chien‐Chen Chou:** Investigation; methodology; formal analysis; resources; data curation; writing—original draft. **Yen‐Cheng Shih:** Investigation; resources; writing—review and editing. **Yi‐Hsiu Chen:** Resources; data curation; writing—review and editing. **Po‐Tso Lin:** Investigation; resources; writing—review and editing. **Chun‐Fu Lin:** Investigation; resources; writing—review and editing. **Po‐Chun Chu:** Software; resources; writing—original draft. **Hao‐Li Liu:** Software; resources; writing—original draft. **Cheng‐Chia Lee:** Conceptualization; investigation; resources; data curation; writing—review and editing. **Hsiang‐Yu Yu:** Conceptualization; investigation; project administration; methodology; formal analysis; resources; data curation; writing—original draft.

## FUNDING INFORMATION

This study was supported by NaviFUS Corp. and Taiwan National Health Research Institutes (NHRI‐EX111‐10905NI, NHRI‐EX112‐11229NI).

## CONFLICT OF INTEREST STATEMENT

H.‐L.L. served as a technical consultant of NaviFUS Corp., Taiwan and currently holds a number of therapeutic ultrasound related patents. P‐C.C. concurrently served as a part‐time Research and Development scientist of NaviFUS Corp., Taiwan. The remaining authors have no conflicts of interest. We confirm that we have read the Journal's position on issues involved in ethical publication and affirm that this report is consistent with those guidelines.

## Supporting information


**Table S1.** Inclusion and exclusion criteria for the clinical trial.

## Data Availability

The data that support the findings of this study are available from the corresponding author upon reasonable request.
