# Supplementary material for: Low‐intensity focused ultrasound neuromodulation for drug‐resistant epilepsy: A randomized, sham‐controlled crossover trial
Source: Epilepsia. 2026 Aug 1;67(11):5715–28. doi: 10.1002/epi.70404 (PMC13768886; doi:10.1002/epi.70404)
Supplement: Supplementary file 1 [file EPI-67-5715-s001.docx]

Table S1. Inclusion and exclusion criteria for the clinical trial

| Inclusion | Exclusion |
| --- | --- |
| Male or female patients aged>= 20 years old. | Patients with concurrent active psychiatric or mood disorders that in the opinion of the investigator would interfere with participation in the study. |
| Patients with drug-resistant epilepsy (defined as at least 3 ASM failed) and 1-4 ASM at the time of study entry. | Presence of pacemakers, implantable cardioverter-defibrillator (ICD), permanent medication pumps, cochlear implants, or deep brain stimulation (DBS) |
| An epileptogenic foci is determined by comprehensive presurgical evaluation. | The skull bone area traversed by the sonication pathway is covered by scars, scalp disorders (e.g., eczema), or atrophy of the scalp. |
| Seizure number is countable and had been recorded at least 6 times within 2-month medical history before screening period. | Clips or other metallic implanted objects in the FUS exposure path, except shunts. |
| Seizure number had been recorded at least 3 times within 28-day screening period. | Abnormal coagulation profile:  a. Platelet (PLT) < 100,000/μL.  b. prothrombin time (PT) >14 sec.  c. activated partial thromboplastin time (APTT) >36 sec.  d. and international normalized ratio (INR) > 1.3. |
| Willing and able to sign written informed consent and be able to comply with the study protocol during the study period | Pregnant or breast-feeding women. |
|  | Coexisting medical problems of sufficient severity to limit compliance with the study. |
|  | Known sensitivity/allergy to Magnetic Resonance Imaging (MRI) contrast agents or any of its components. |
|  | Use of any recreational drugs or history of drug addiction or known history of substance or alcohol abuse. |
|  | Any other condition that, in the investigator’s judgment, might affect study endpoints or might increase the risk to the patients or decrease the chance of obtaining satisfactory data needed to achieve the objectives of the study. |
|  | Any ASM treatment change during the baseline (screening period) |
|  | Vagus nerve stimulation (VNS) dosing changes within 2 months before baseline (screening period). |
|  | Radiofrequency thermocoagulation (RFTC) within 2 months before baseline (screening period). |
|  | Patients has participated other clinical trial within 4 weeks of entering this study. |
